# Trialling of an optimal health programme (OHP) across chronic disease

**DOI:** 10.1186/s13063-016-1560-5

**Published:** 2016-09-09

**Authors:** Chantal F. Ski, David R. Thompson, David J. Castle

**Affiliations:** 1 Centre for the Heart and Mind, Australian Catholic University, Melbourne, VIC 3000 Australia; 2 Department of Psychiatry, University of Melbourne, Melbourne, VIC 3010 Australia; 3 Mental Health Service, St. Vincent’s Hospital, Melbourne, VIC 3065 Australia

**Keywords:** Collaborative therapy, Cost-effectiveness, Chronic disease, Psychoeducational, Psychosocial, Randomised controlled trial

## Abstract

Population ageing is a worldwide phenomenon, most advanced in developed countries and expected to continue over the next few decades. As people are surviving longer with age-associated disease and disability, there is an imperative to identify innovative solutions for an already overburdened health care system. Such innovations need to be focused on disease management, taking into consideration the strong associations that have been established between psychosocial factors and pathophysiological mechanisms associated with chronic disease. Aside from personal and community costs, chronic diseases produce a significant economic burden due to the culmination of health care costs and lost productivity. This commentary reports on a programme of research, Translating Research, Integrated Public Health Outcomes and Delivery, which will evaluate an optimal health programme that adopts a person-centred approach and engages collaborative therapy to educate, support and improve the psychosocial health of those with chronic disease. The effectiveness of the optimal health programme will be evaluated across three of the most significant contributors to disease burden: diabetes mellitus, chronic kidney disease and stroke. Cost-effectiveness will also be evaluated. The findings derived from this series of randomised controlled trials will also provide evidence attesting to the potential applicability of the optimal health programme in other chronic conditions.

## Background

Over the past few decades, tremendous advances in medical science and technology have enhanced the understanding and treatment of chronic diseases and led to increased longevity [1]. In spite of this, chronic diseases remain the leading cause of illness, disability and death, not only in Australia, accounting for 90 % of all deaths in 2011 [2], but also worldwide [3]. Given the enormous personal, social and economic impact of chronic diseases, tackling them has emerged as the predominant challenge to global health [4]. It is well established that psychosocial factors contribute significantly to the aetiology, development and trajectory of chronic diseases, as well as to quality of life [5, 6]. Even so, far too often psychosocial aspects of chronic diseases are overlooked [7]. In a time when medical breakthroughs equate to increased human lifespan, essential now are effective (including cost-effective) and sustainable interventions that target the *management* of chronic disease.

An optimal health programme (OHP) that adopts a person-centred approach and comprises psychosocial supports and a skill base designed to maintain optimal chronic disease management was originally designed to support patients with mental illness [8, 9]. The OHP is based on a collaborative therapy framework sensitive to service structure, resources and staff mix to ensure maximum likelihood of integration into existing health services [10]. With the intention of enhancing self-efficacy, self-management, care coordination and quality of life the OHP has been adapted to the broader context of chronic physical diseases. This commentary supplements a series of study protocols of randomised controlled trials that will evaluate the OHP across three of the most significant contributors to disease burden: diabetes mellitus (DM), chronic kidney disease (CKD) and stroke [11].

### The chronic disease challenge

It is widely acknowledged that many chronic diseases, particularly lifestyle-related diseases, share similarities in their underlying aetiology, risk factors and progression [12]. Epidemiological studies have provided evidence of strong associations between chronic physical illness and an increased risk of mental illness [13, 14]. For example, links have been identified between anxiety and depression and DM [15], CKD [16] and stroke [17]. Recognition of these parallels and commonalities provides merit for a holistic approach to chronic disease management that targets psychosocial empowerment, collaborative care and health service integration—the primary foci of the OHP.

### Diabetes mellitus

The prevalence of DM is reported to have reached epidemic proportions [18, 19]. The management of DM imposes a complex self-care regimen that can be difficult to sustain, including healthy eating, regular physical activity, taking medications as recommended, checking blood glucose levels and attending medical appointments. Owing to the evolving nature of DM and the complex interactions it involves between physiological, psychological and environmental factors, DM is acknowledged to be one of the most challenging chronic diseases [20, 21]. Recent guidelines have recommended a collaborative approach to be delivered by a multidisciplinary team that tailors interventions to each individual’s situation [22]. Thus, interventions involving shared decision-making, pragmatic problem-solving and promotion of behaviour change strategies should be considered when working toward achieving sustainable self-management of DM.

### Chronic kidney disease

The past two decades have seen a rapid escalation in the prevalence of CKD due to an ageing population and a growing prevalence of diabetes, hypertension and obesity [23]. Recent estimates indicate that the cumulative costs of providing treatment to new and existing patients in Australia from 2009 to 2020 will reach $12 billion [24]. A recent review of CKD self-management programmes concluded that life contexts, socioeconomic factors, health literacy and psychological factors, as well as communication with health care providers, all contribute to an individual’s adherence to treatment, and further that self-efficacy skill-building could potentially improve treatment adherence [25]. Of note, CKD is largely a preventable chronic condition, as many of its risk factors are modifiable, including high blood pressure, tobacco smoking, physical inactivity, poor nutrition and obesity [24].

### Stroke

Stroke is the second leading cause of death worldwide (11 %) and is often associated with long-term disability [26]. The abrupt onset of stroke and its numerous serious sequelae profoundly affect the psychological health and quality of life of stroke survivors and their carers [27, 28]. Role reversals and unexpected physical, cognitive and emotional demands have been demonstrated to contribute to adverse outcomes such as anxiety, depression, cardiovascular disease and mortality for both survivors and carers [27–29]. Only recently has research in the field of stroke shifted from a physical emphasis to include psychological and social elements with a focus on carers; yet, the stroke survivor-carer dyad has received minimal attention [30]. In recognition of the indispensable role of caregivers in the management and co-ordination of care, often at the expense of their own health, the stroke OHP has been developed in consideration of the needs of both stroke survivors and their carers.

### The Optimal Health Program (OHP)

**Fig. 1:**
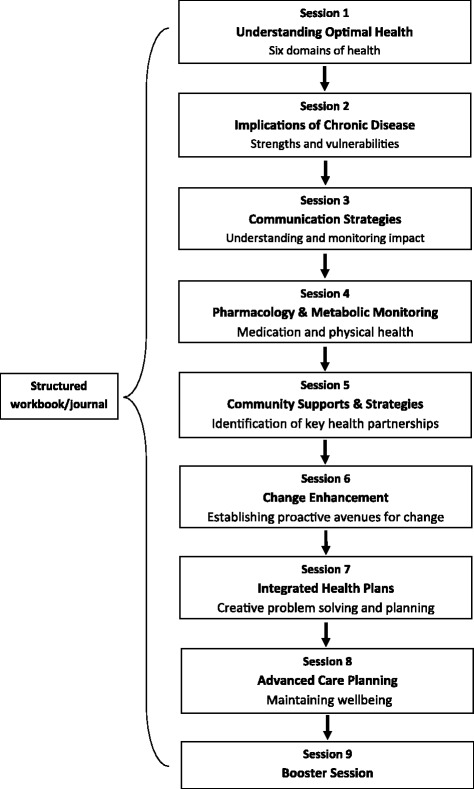
Translating Research, Integrated Public Health Outcomes and Delivery (TRIPOD) generic optimal health programme

This series of randomised controlled trials compose the Translating Research, Integrated Public Health Outcomes and Delivery (TRIPOD) programme of research. Within TRIPOD, the OHP embraces a modular format that has been tailored specifically to various stages of each of the three chronic diseases, engaging consumers and clinicians from acute care through to community. Figure 1 presents a generic diagram of the OHP; descriptions of OHP modules specific to DM, CKD and stroke are presented in each of the respective protocol papers. In brief, the modules delivered by health care facilitators (e.g., nurse, psychologist) are based on a structured workbook/journal, conducted weekly and approximately 1 h in duration. Session 1 explores chronic disease and self-management from a holistic perspective, incorporating social, physical, emotional, intellectual, employment and spiritual aspects. Sessions 2 and 3 see the introduction of a health plan involving psychosocial implications and engagement with health care providers. Session 4 is focused on medication management and physical health. Session 5 helps to identify and include key community supports. Session 6 establishes new proactive avenues for change, and session 7 integrates these via goal-setting around the complexities of chronic disease. Session 8 strategizes sustainable advance care planning. A ‘booster session’ is run 3 months after session 8 to review progress against health plans.

When delivering the OHP, emphasis is placed on a collaborative partnership between the facilitator and the participant, who work together to develop viable health action plans and to discuss and arrange referrals with the multidisciplinary team, dependent on ongoing caregiver needs. The facilitator encourages the participant to identify early warning signs of stress and illness and provides them with the skills to integrate healthy coping strategies to successfully manage stressful periods. Importantly, enhancing self-efficacy is at the centre of the programme with the aim of improving self-management skills, optimising psychosocial health and increasing the capacity to access key supports. By providing enablers for increased independence and empowerment, we envisage that users of the OHP will work towards a shift in focus from the person being ‘dependent on’ services to being ‘supported by’ services.

## Conclusions

The devastating effects of chronic disease not only impact upon public health but also cause much harm to the socioeconomic fabric of communities [31]. In the absence of evidence-based and cost-effects models for the management of chronic diseases, the human, social and economic costs will continue to escalate and overwhelm the capacity to address them [32]. Furthermore, the growing understanding that many chronic diseases arise from similar underlying causes and have similar features challenges health care professionals to transform the way in which they respond to management of these diseases. In this series of randomised controlled trials, we will adapt a generic OHP for three chronic diseases known to have the greatest impact on disease burden: DM, CKD and stroke. In doing so, we will attempt to address the challenge of effective and sustainable chronic disease management via an OHP that (1) has been developed in close collaboration with health care clinicians, patients and carers; (2) addresses psychological, social and physiological aspects of care; (3) provides enablers for increased independence and empowerment; and (4) incorporates a comprehensive health economic cost analysis. The findings derived from these trials will provide evidence for the efficacy and feasibility of our OHP to be delivered across a far greater range of chronic conditions.

## Abbreviations

CKD: Chronic kidney disease
DM: Diabetes mellitus
OHP: Optimal Health Program
TRIPOD: Translating Research, Integrated Public Health Outcomes and Delivery

## References

[1] Lichtenberg FR (2009). The quality of medical care, behavioral risk factors, and longevity growth. Int J Health Care Finance Econ.

[2] Australian Institute of Health and Welfare (AIHW). Key indicators of progress for chronic disease and associated determinants: data report. Catalogue number PHE 142. Canberra, Australia: AIHW; June 2011. http://www.aihw.gov.au/WorkArea/DownloadAsset.aspx?id=10737419243&libID=10737419242. Accessed 30 Aug 2016.

[3] Global Burden of Disease Study 2013 Collaborators (2015). Global, regional, and national incidence, prevalence, and years lived with disability for 301 acute and chronic diseases and injuries in 188 countries, 1990–2013: a systematic analysis for the Global Burden of Disease Study 2013. Lancet.

[4] Bauer UE, Briss PA, Goodman RA, Bowman BA (2014). Prevention of chronic disease in the 21st century: elimination of the leading preventable causes of premature death and disability in the USA. Lancet.

[5] Schneiderman N, Antoni MH, Saab PG, Ironson G (2001). Health psychology: psychosocial and biobehavioral aspects of chronic disease management. Annu Rev Psychol.

[6] Tran MW, Weiland TJ, Phillips GA (2015). Psychosocial and nonclinical factors predicting hospital utilization in patients of a chronic disease management program: a prospective observational study. J Ambul Care Manage.

[7] Perruccio AV, Katz JN, Losina E (2012). Health burden in chronic disease: multimorbidity is associated with self-rated health more than medical comorbidity alone. J Clin Epidemiol.

[8] Castle D, White C, Chamberlain J, Berk M, Berk L, Lauder S (2010). Group-based psychosocial intervention for bipolar disorder: randomised controlled trial. Br J Psychiatry.

[9] Gilbert MM, Chamberlain JA, White CR, Mayers PW, Pawsey B, Liew D (2012). Controlled clinical trial of a self-management program for people with mental illness in an adult mental health service – the Optimal Health Program (OHP). Aust Health Rev.

[10] Castle DJ, Gilbert M (2006). Collaborative therapy: framework for mental health. Br J Psychiatry.

[11] World Health Organisation (WHO) (2011). Global status report on noncommunicable diseases 2010.

[12] Sagner M, Katz D, Egger G, Lianov L, Schulz KH, Braman M (2014). Lifestyle medicine potential for reversing a world of chronic disease epidemics: from cell to community. Int J Clin Pract.

[13] Chapman DP, Perry GS, Strine TW (2005). The vital link between chronic disease and depressive disorders. Prev Chronic Dis [serial online].

[14] Di Benedetto M, Lindner H, Aucote H, Churcher J, McKenzie S, Croning N (2014). Co-morbid depression and chronic illness related to coping and physical and mental health status. Psychol Health Med.

[15] de Groot M, Anderson R, Freedland KE, Clouse RE, Lustman PJ (2001). Association of depression and diabetes complications: a meta-analysis. Psychosom Med.

[16] Hedayati SS, Finkelstein FO (2009). Epidemiology, diagnosis, and management of depression in patients with CKD. Am J Kidney Dis.

[17] Whyte EM, Mulsant BH (2002). Post stroke depression: epidemiology, pathophysiology, and biological treatment. Biol Psychiatry.

[18] Ginter E, Simko V (2012). Type 2 diabetes mellitus, pandemic in 21st century. Adv Exp Med Biol.

[19] Shaw JE, Sicree RA, Zimmet PZ (2010). Global estimates of the prevalence of diabetes for 2010 and 2030. Diabetes Res Clin Pract.

[20] Long GH, Johansson I, Rolandsson O, Wennberg P, Fharm E, Weinehall L (2015). Healthy behaviours and 10-year incidence of diabetes: a population cohort study. Prev Med.

[21] Soni A, Ng SM (2014). Intensive diabetes management and goal setting are key aspects of improving metabolic control in children and young people with type 1 diabetes mellitus. World J Diabetes.

[22] Craig ME, Twigg SM, Donaghue KC, Cheung NW, Cameron FJ, Conn J (2011). Australian Type 1 Diabetes Guidelines Expert Advisory Group. National evidence-based clinical care guidelines for type 1 diabetes in children, adolescents and adults.

[23] Chen T, Harris DC (2015). Challenges of chronic kidney disease prevention. Med J Austral.

[24] Australian Institute of Health and Welfare (AIHW) (2014). Projections of the prevalence of treated end-stage kidney disease in Australia 2012–2020. Catalogue number PHE 176.

[25] Bonner A, Havas K, Douglas C, Thepha T, Bennett P, Clark R (2014). Self-management programmes in stages 1–4 chronic kidney disease: a literature review. J Ren Care.

[26] Mendis S, Puska P, Norrving B (2011). Global atlas on cardiovascular disease prevention and control.

[27] Ski C, O’Connell B (2007). Stroke: the increasing complexity of carer needs. J Neurosci Nurs.

[28] Kniepmann K (2012). Female family carers for survivors of stroke: occupational loss and quality of life. Br J Occup Ther.

[29] Kirkevold M, Bronken BA, Martinsen R, Kvigne K (2012). Promoting psychosocial well-being following a stroke: developing a theoretically and empirically sound complex intervention. Int J Nurs Stud.

[30] Godwin KM, Ostwald SK, Cron SG, Wasserman J (2013). Long-term health-related quality of life of stroke survivors and their spousal caregivers. J Neurosci Nurs.

[31] Hunter DJ, Reddy KS (2013). Noncommunicable diseases. N Engl J Med.

[32] Arredondo A, Aviles R, Clement F (2015). Costs and Epidemiological Changes of Chronic Diseases: Implications and Challenges for Health Systems. PLoS One.

